# Supplementary material for: BANDITS: Bayesian differential splicing accounting for sample-to-sample variability and mapping uncertainty
Source: Genome Biol. 2020 Mar 16;21:69. doi: 10.1186/s13059-020-01967-8 (PMC7075019; doi:10.1186/s13059-020-01967-8)
Supplement: Supplementary file 2 — Additional file 2 Review history. [file 13059_2020_1967_MOESM2_ESM.docx]

**Review History**

**First round of review**

**Reviewer 1**

**Comments to author:**

This paper presents a new method for differential transcript usage analysis and compares its performance to existing tools using simulated and experimental RNA-seq data.
Overall the paper is clearly written and provides an interesting tool with superior or similar performance to its competitors in detecting differential transcript usage. It would generally benefit from further analysis to demonstrate the advantages of using this tool to the wide readership of Genome Biology.

Major points:
1) The title suggests BANDITS is able to account for "sample-to-sample variability and mapping uncertainty". However no data are shown to support this claim, especially in comparison to the competitors. Only batch effect is mentioned in the discussion section without showing data, and other sources of sample variation such as biological differences are ignored. Mapping uncertainty related to multiple isoforms from the same gene is introduced, but uncertainty related to homologous genes or pseudogenes is not discussed. Examples should be included supporting these capacities from available experimental datasets in a figure or supp figure of the article.

2) The article states that BANDITS is not limited to only comparisons of two groups of samples, which is an important advantage of the method. However, this capacity is never shown using available datasets and tested against other methods.

3) The article would be stronger and would interest a wider readership if an example was given showing novel biological insight gained using BANDITS. This could be done by re-analysing an available dataset.

4) The "median position" in Table 1 needs to be justified as a measure of performance. The interpretation of this result depends on whether the validated events provide a general profile or present only a group of events / genes with a special feature. In the latter case, a tool capturing that feature and ignoring everything else will produce a low "median position" result but is not suitable for general analysis.

5) Isoforms can rise from alternative transcription start sites and terminal sites in addition to splicing. Thus, isoform expression / usage quantification is not entirely about splicing. This should be reflected in the text.

6) For the study to be reproducible, it would be important to include all parameter values for all tools used including to generate the simulation data, and to process the reads from Best dataset.

Minor points:
1) Why is hg38 used to analyse the experimental data and hg37 used for the simulation? The use of hg37 should be justified.

2) In the Abstract: 'Here we present BANDITS, a R/Bioconductor package to perform differential splicing, at both gene and transcript-level, based on RNA-seq data. ' A word is missing after splicing, perhaps 'analysis'?

3) The exact same figure legends are used for Figures 1 and S1 as well as for Figure 2 and S2. While it is possible to obtain information about these pairs of figures in the main text, a bit more information in the legend would be helpful for the readers. The legends of Figure S1 and S2 could refer to Figures 1 and 2 respectively.

4) Figure 2C: why are not all methods present?

**Reviewer 2**

**Comments to author:**

In this manuscript, Tiberi and Robinson present a new method to identify differential transcript usage (DTU) in RNA-Seq data called BANDITS.  The distinguishing feature of this method is the use of a Bayesian hierarchical model with a Dirichlet multinomial structure, which allows variance between biological replicates to be modeled.  Variance from biological replication is often inadequately addressed in differential splicing analysis, so this point is a very useful feature.  The tool requires equivalence classes counts as input, which requires a third-party method to calculate, but is flexible in allowing input from more than one tool.

The software is available as a Bioconductor package, with documentation and instructions provided.  Results in the Bioconductor vignette are reproducible.  The authors describe a useful new tool for the community.  They have demonstrated that BANDITS performs well in regards to type I/II error and in compute time.   However, in my opinion the manuscript requires some additional biological context and applicability beyond these quantitative benchmarks.

Major points:

The relationship of DTU to alternative splicing is a little ambiguous. I understand the target of this approach is not canonical splicing events, but does it consider whether or not the isoforms derive from the same parent transcript?  Do all DTU instances identified derive from splicing differences?  A brief clarification along these lines would be helpful. On p.8,col.1,line 21, the phrase 'BANDITS targets splicing genes and transcripts' is unclear.

An example of real RNA-Seq results from an annotated gene is important to add biological perspective.  This may be as simple as displaying browser tracks along with the corresponding proportion plot similar to what is shown in the vignette.

Minor points:

The authors state that a feature for this tool is the ability to compare more than two groups.  However, I do not see this demonstrated.  An example should be provided in the manuscript or Bioconductor vignette.

Some methodological detail is provided in Github and/or Figshare, but for convenience in a manuscript, it should be provided in brief. For example, the simulation approach appears to based on RSEM and used in a previous publication. To aid the reader, the method can be described in brief so that the reader doesn't have to search for it.

Figure 2C: Please explain why results for rats are present in 2A-B but not in 2C

Please provide a little more detail on the compute time benchmarking, such as the available RAM and which tools used parallelization if this is known.

**Reviewer 3**

This study presents a model for inferring Differential Transcript Usage (DTU) given replicated RNA-seq data samples. The authors propose a novel Bayesian model which jointly treats read mapping uncertainty and transcript expression counts as latent variables, also allowing for within-replicate variability.

Assigning an informative prior distribution on model parameters, the posterior distribution is inferred using MCMC. In order to test for DTU, the posterior distribution is approximated by a (multivariate) normal distribution with parameters inferred from the MCMC sample. Finally, DTU is tested via the Wald statistic. The contributed method is benchmarked against alternative approaches using simulation studies and experimental datasets.

The topic is important and the proposed approach is interesting. The presentation is easy to follow. The paper is a mix of methodological development, efficient computational considerations and contribution of an open source software package. As such I feel the paper is suitable for publication in Genome Biology, although I do have some concerns, as detailed below.

**1 Specific comments**

1.1 **The prior on δ**

The default prior (pages 3-4) on the parameters of the Dirichlet distribution is based on data pre-processing using another quantification tool (DRIMSeq). Although the authors do mention that δks could follow vaguely informative priors, I’ve found the adopted approach overly informative. Ideally, the authors should include in all of their comparisons and reported results (including diagrams and tables) the results of BANDITS arising from vaguely informative priors. This will give an idea to the reader about the sensitivity of the method with respect to prior choices.

1.2 **Normalization with respect to effective length**

The authors state that using the specific normalization (that is, using π¯ T k ’s instead of π¯k’s) leads to improved performance when testing for DTU. Since all other methods are actually testing for differences in the latter, it would make sense to evaluate all methods on a common basis, that is, π¯k’s. This will give a precise idea about the ability of each method to detect differences on a commonly used definition of what constitutes a ‘‘difference’’. Then, the authors can argue about using the specific normalization based on π¯ T k ’s, focusing on the enhanced performance of their proposed method.

**2 Minor comments**

**2.1** **Transcript pre-filtering**

The authors propose to use estimated counts in order to filter low expressed transcripts. Could you comment on the specific quantification choice (Salmon)? For example: how sensitive are the results if another quantification tool is used?

**2.2** **Computational benchmark**

Briefly discuss the amount of memory used by each method

**Authors Response**

**Point-by-point responses to the reviewers’ comments:**

Reviewer #1: This paper presents a new method for differential transcript usage analysis and compares its performance to existing tools using simulated and experimental RNA-seq data.

Overall the paper is clearly written and provides an interesting tool with superior or similar performance to its competitors in detecting differential transcript usage. It would generally benefit from further analysis to demonstrate the advantages of using this tool to the wide readership of Genome Biology.

Major points: 1) The title suggests BANDITS is able to account for "sample-to-sample variability and mapping uncertainty". However no data are shown to support this claim, especially in comparison to the competitors. Only batch effect is mentioned in the discussion section without showing data, and other sources of sample variation such as biological differences are ignored. Mapping uncertainty related to multiple isoforms from the same gene is introduced, but uncertainty related to homologous genes or pseudogenes is not discussed. Examples should be included supporting these capacities from available experimental datasets in a figure or supp figure of the article.

*REPLY: Thank you for your comment.*

*We do not mean to state that BANDITS can deal with all sources of variability related to biological and measurement processes; we specifically refer to two such sources of uncertainty. With “sample-to-sample” variabilty, we mean that, via a Dirichlet-multinomial hierarchical model, we allow each sample to have a distinct set of “pi” parameters; in other words, the transcript relative abundance is allowed to vary from individual to individual due to biological variability.*

*With “mapping uncertainty”, we refer to the uncertainty generated when reads are compatible with multiple transcripts; in these cases, the transcript of origin of each read is unknown and the transcript (relative and total) abundance cannot be computed exactly. To deal with this source of uncertainty, we treat the transcript of origin of each read as a latent variable (i.e., an unknown parameter) that we sample in our model. Note that Supplementary Table S1 lists which one of these two sources of variability is dealt with by each method.*

*Concerning homologous genes and pseudogenes: all annotated transcripts are treated identically. Therefore, if transcripts from homologous genes and pseudogenes are included in the transcriptome provided, the mapping uncertainty (arising from reads compatible with multiple transcripts) will be modelled by BANDITS as usual. We added two comments to clarify this issue at page 2, right column, lines 25-27 and 32-36.*

*Moreover, an indirect evaluation of the improved performance that these two features lead to can be obtained by comparing our method to DRIMSeq and cjBitSeq, which are the most similar models to BANDITS.*

*DRIMSeq accounts for the sample-to-sample variability via a Dirichlet-multinomial model (the same model used by BANDITS), but it neglects the mapping uncertainty (it inputs estimated counts).*

*cjBitSeq, similarly to BANDITS, uses a latent variable structure to account for the mapping uncertainty (by mapping what transcript reads arose from), but it does not allow for sample-specific parameters.*

*In all simulated and experimental data analyses, BANDITS leads to higher performance compared to DRIMSeq and cjBitSeq, which suggests that each one of the two features considered (sample-to-sample variability and mapping uncertainty) provides improved detection of differential transcript usage.*

2) The article states that BANDITS is not limited to only comparisons of two groups of samples, which is an important advantage of the method. However, this capacity is never shown using available datasets and tested against other methods.

*REPLY: The same issue was also raised by reviewer 2 (minor point 1). We have added a new simulation study based on a 3-group comparison. We have recycled the 6 vs. 6 simulation study by separating the first group into two sub-groups of 3 samples each, hence obtaining a 3 vs. 3 vs. 6 structure. We have benchmarked BANDITS and the other methods that allow to jointly test more than 2 groups (DRIMSeq and the three variants of DEXSeq), please see page 6, right column, lines 1-16 and Supplementary Figures S10-S11. Furthermore, to illustrate the usage of BANDITS on a multi-group comparison, we have also updated the vignette of BANDITS (Bioconductor devel, release 11, https://bioconductor.org/packages/devel/bioc/vignettes/BANDITS/inst/doc/BANDITS.ht ml).*

3) The article would be stronger and would interest a wider readership if an example was given showing novel biological insight gained using BANDITS. This could be done by re-analysing an available dataset.

*REPLY: The main goal here is to present a novel methodological framework with an appropriate benchmarking against the main competitors in the field; therefore, the manuscript focuses on demonstrating the method, rather than on providing new biological insight. Also, note that “A Method article need not necessarily provide novel biological insights” (*[*https://genomebiology.biomedcentral.com/submissionguidelines/preparing-your-manuscript/method*](https://genomebiology.biomedcentral.com/submissionguidelines/preparing-your-manuscript/method)*).*

*Nevertheless, to show that BANDITS can be used to identify new discoveries, we added a further analysis from the “Best et al.” dataset. We considered the top 100 and top 200 ranked genes from each method (ranked according to statistical significance), and counted the number of validated genes in each set.*

*We found that, in both sets, BANDITS top ranked genes contain more validated genes than any other method: 18/100 and 25/200, while the sets defined by the other methods contain between 3 and 14/100 and between 9 and 20/200. This shows the ability of BANDITS to retrieve more validated differentially spliced genes than any other method.*

*This shows the ability of BANDITS to retrieve more validated differentially spliced genes than any other method.*

*We further performed a gene ontology (GO) analysis to test the ability of methods to retrieve enriched GO terms. We first tested GO terms from the 82 validated genes and found 338 enriched GO terms with a p-value < 0.01 (out of a total of 22,911), which we define our ‘validated’ set of GO terms. We then tested GO terms from the results of each method and selected the top 338 enriched GO terms (as many as in the ‘validated’ set). When considering FDR thresholds 0.05 and 0.01, BANDITS and BANDITS_inv recover between 32 and 35% of the “validated” GO terms, while for the remaining methods this ratio ranges between 14 and 35%. Overall, this demonstrates that BANDITS can be used to accurately gain new biological insight.*

*All new results have been added to Table 1 (at page 15) and are illustrated at page 6, right column, lines 34-36 and at page 7, left column, lines 5-16. Furthermore, in response to major point 2 from Reviewer 2 (which we think is related to this issue), we have also added an in depth visual inspection of two individual genes found from BANDITS when analyzing the the “Best el al.” dataset. These two examples show how BANDITS can be used to identify differentially spliced genes as well as the individual transcripts involved in the splicing difference (please see our response below for more details). This new analysis is illustrated in the main text (page 7, left column, lines 17-23) and in Supplementary Section S1.5, Supplementary Tables S9-S10 and Supplementary Figures S13-S17.*

4) The "median position" in Table 1 needs to be justified as a measure of performance. The interpretation of this result depends on whether the validated events provide a general profile or present only a group of events / genes with a special feature. In the latter case, a tool capturing that feature and ignoring everything else will produce a low "median position" result but is not suitable for general analysis.

*REPLY: To provide alternative measures, we now added to Table 1 (at page 15) the number of validated genes in the top 100 and 200 ranked genes as well as the mean number of “validated” GO terms for FDR thresholds 0.01 and 0.05 (see major point 3 above). We think median position of validated genes is a reasonable measure of how accurately genes are ranked, but we do not uniquely rely on it in our judgment. Indeed, results of the “Best el al.” analysis are jointly summarized by the ROC curve (Figure 3) and (now) 8 quantitative measures reported in Table 1 (median position of validated genes, number of validated genes in top 100 and top 200 ranked genes, AUC and partial AUCs of level 0.1 and 0.2, mean number of recovered “validated” GO terms for FDR thresholds 0.01 and 0.05).*

5) Isoforms can rise from alternative transcription start sites and terminal sites in addition to splicing. Thus, isoform expression / usage quantification is not entirely about splicing. This should be reflected in the text.

*REPLY: We feel major point 5 from Reviewer 1 and major point 1 from Reviewer 2 are highly connected, so decided to answer both together. Differential transcript usage and differential transcript expression methods target differences in annotated transcripts, which may arise due to differential splicing as well as alternative start and terminal sites of the same transcript; all such approaches (including differential exon usage) cannot distinguish between the two sources of the difference. Thanks for raising this issue: we clarified this aspect in the main text at page 1, right column, lines 34-38.*

6) For the study to be reproducible, it would be important to include all parameter values for all tools used including to generate the simulation data, and to process the reads from Best dataset.

*REPLY: The script to run all analyses are reported in a publicly available Github repository: https://github.com/SimoneTiberi/BANDITS_manuscript. The scripts are organized in four folders: 1) “Simulated data 6 vs 6” contains the code to generate the new simulated data;*

*2) “Alignment and quantification” has the scripts to align and quantify reads (from all simulated and experimental datasets);*

*3) “Methods” contains the code to run all differential methods;*

*4) “Plots and Tables” has the script to generate the Figures and Tables presented in both in the main manuscript and Supplementary text. The link to the repository is reported in sub-section “Availability of data and material” at page 14, left column, line 39.*

Minor points:

1) Why is hg38 used to analyse the experimental data and hg37 used for the simulation? The use of hg37 should be justified.

*REPLY: For the experimental data, we relied on the latest available genome at the time, which was hg38.92. The 3 vs. 3 simulated data was taken from Soneson et al. (2016) were reads were simulated using hg37.71. When simulating the 6 vs. 6 simulation study we modified the pipeline in Soneson et al. (2016) and kept the same genome (hg37). Therefore reads from the simulation studies were aligned to hg37 genome. We have added a sentence in the Supplementary material to clarify this ambiguity (first 10 lines of “S1.4 Results details” Section, at the bottom of page 4).*

2) In the Abstract: 'Here we present BANDITS, a R/Bioconductor package to perform differential splicing, at both gene and transcript-level, based on RNA-seq data. ' A word is missing after splicing, perhaps 'analysis'?

*REPLY: Thanks for your comment. We think that both “differential splicing” and “differential splicing analysis” illustrate the type of analysis that BANDITS perform. We choose the former for brevity, which helped us to keep the abstract within Genome Biology word limits.*

3) The exact same figure legends are used for Figures 1 and S1 as well as for Figure 2 and S2. While it is possible to obtain information about these pairs of figures in the main text, a bit more information in the legend would be helpful for the readers. The legends of Figure S1 and S2 could refer to Figures 1 and 2 respectively.

*REPLY: Thank you for your comment. Legends are indeed almost identical (one “gene-level” and one “transcript-level”) because they actually refer to the same plot for gene- and transcript-level tests; so having almost identical legends was intentional. We have extended the information provided in the legends of Figures 1, 2, S1 and S2 (page 14 of the main and 12-13 of the Supplementary material).*

4) Figure 2C: why are not all methods present?

*REPLY: Reviewer 2 also noticed that not all methods were present in Figure 2C (minor point 3). RATs was accidentally removed from Figures 2C and S2C; we edited both Figures to include all methods. Thank you both for noticing this.*

Reviewer #2: In this manuscript, Tiberi and Robinson present a new method to identify differential transcript usage (DTU) in RNA-Seq data called BANDITS. The distinguishing feature of this method is the use of a Bayesian hierarchical model with a Dirichlet multinomial structure, which allows variance between biological replicates to be modeled. Variance from biological replication is often inadequately addressed in differential splicing analysis, so this point is a very useful feature. The tool requires equivalence classes counts as input, which requires a third-party method to calculate but is flexible in allowing input from more than one tool. The software is available as a Bioconductor package, with documentation and instructions provided. Results in the Bioconductor vignette are reproducible. The authors describe a useful new tool for the community. They have demonstrated that BANDITS performs well in regards to type I/II error and in compute time. However, in my opinion the manuscript requires some additional biological context and applicability beyond these quantitative benchmarks.

Major points:

1) The relationship of DTU to alternative splicing is a little ambiguous. I understand the target of this approach is not canonical splicing events, but does it consider whether or not the isoforms derive from the same parent transcript? Do all DTU instances identified derive from splicing differences? A brief clarification along these lines would be helpful. On p.8,col.1,line 21, the phrase 'BANDITS targets splicing genes and transcripts' is unclear.

*REPLY: We feel major point 5 from Reviewer 1 and major point 1 from Reviewer 2 are highly connected, so decided to answer both together.*

*Differential transcript usage and differential transcript expression methods target differences in annotated transcripts, which may arise due to differential splicing as well as alternative start and terminal sites of the same transcript; all such approaches (including differential exon usage) cannot distinguish between the two sources of the difference.*

*Thanks for raising this issue: we clarified this aspect in the main text at page 1, right column, lines 34-38.*

*We have also modified the sentence “BANDITS targets splicing genes and transcripts” to: “BANDITS targets genes and transcripts undergoing differential splicing” (page 9, left column, lines 19-21).*

2) An example of real RNA-Seq results from an annotated gene is important to add biological perspective. This may be as simple as displaying browser tracks along with the corresponding proportion plot similar to what is shown in the vignette.

*REPLY: From the “Best el al.” data analysis, we considered two genes selected from BANDITS (with adjusted p-value 0.000), one of which belonged to the set of validated genes, and one not previously validated. For both genes, we report BANDITS test results, display transcript-level proportions estimated from BANDITS and show the sample-specific coverage and junction tracks via IGV software. BANDITS proportion plots indicate clear differences between groups in differentially spliced transcripts; some of these differences can also be clearly visualized on the IGV plots, particularly for transcripts ENST00000524595 and ENST00000521071, which are almost only expressed in one group. These examples show how BANDITS can be effectively used to identify differentially spliced genes as well as the involved transcripts. This new analysis is illustrated in the main text (page 7, left column, lines 17-23) and in Supplementary Section S1.5, Supplementary Tables S9-S10 and Figures S13-S17.*

*Furthermore, in response to major point 3 from Reviewer 1 (which is partially connected to this issue), we have also added further analyses from “Best el al.” dataset to illustrate how BANDITS can identify more validated genes and more sensible gene ontology terms compared to other methods (please see above for more details); these analyses are described at page 6, right column, lines 34-36 and at page 7, left column, lines 5-16.*

Minor points:

1) The authors state that a feature for this tool is the ability to compare more than two groups. However, I do not see this demonstrated. An example should be provided in the manuscript or Bioconductor vignette.

*REPLY: Reviewer 1 also raised this issue (major point 2): we report the same answer written above. We have added a new simulation study based on a 3-group comparison. We have recycled the 6 vs. 6 simulation study by separating the first group into two sub-groups of 3 samples each, hence obtaining a 3 vs. 3 vs. 6 structure. We have benchmarked BANDITS and the other methods that allow to jointly test more than 2 groups (DRIMSeq and the three variants of DEXSeq), please see page 6, right column, lines 1-16 and Supplementary Figures S10-S11. Furthermore, to illustrate the usage of BANDITS on a multi-group comparison, we have also updated the vignette of BANDITS (Bioconductor devel, release 11, https://bioconductor.org/packages/devel/bioc/vignettes/BANDITS/inst/doc/BANDITS.ht ml).*

2) Some methodological detail is provided in Github and/or Figshare, but for convenience in a manuscript, it should be provided in brief. For example, the simulation approach appears to based on RSEM and used in a previous publication. To aid the reader, the method can be described in brief so that the reader doesn't have to search for it.

*REPLY: Thanks for your comments. We have added a new sub-section (Simulation) in Methods to provide more details about the simulation pipelines (page 12, lines 17-39 of the left column and lines 1-13 of the right column).*

3) Figure 2C: Please explain why results for rats are present in 2A-B but not in 2C.

*REPLY: Reviewer 1 also noticed that not all methods were present in Figure 2C (minor point 4); again, we report the same answer we wrote above. RATs was accidentally removed from Figures 2C and S2C; we edited both Figures to include all methods. Thank you both for noticing this.*

4) Please provide a little more detail on the compute time benchmarking, such as the available RAM and which tools used parallelization if this is known.

*REPLY: This issue was also raised by Reviewer 3 (minor point 2.2). To address this, we have extended our computational benchmark to also measure the maximum RAM used by each method. The new results are described in the main (page 8, left column, lines 4-13), and shown in Supplementary Table S8 and Figure S12.*

Reviewer #3: This study presents a model for inferring Differential Transcript Usage (DTU) given replicated RNA-seq data samples. The authors propose a novel Bayesian model which jointly treats read mapping uncertainty and transcript expression counts as latent variables, also allowing for within-replicate variability.

Assigning an informative prior distribution on model parameters, the posterior distribution is inferred using MCMC. In order to test for DTU, the posterior dis- tribution is approximated by a (multivariate) normal distribution with parameters inferred from the MCMC sample. Finally, DTU is tested via the Wald statistic. The contributed method is benchmarked against alternative approaches using simula- tion studies and experimental datasets.

The topic is important and the proposed approach is interesting. The presenta- tion is easy to follow. The paper is a mix of methodological development, efficient computational considerations and contribution of an open source software package. As such I feel the paper is suitable for publication in Genome Biology, although I do have some concerns, as detailed below.

*REPLY: We have created a new Section in the main text, called Sensitivity analyses (page 8, right column), where we present results from major points 1 (prior on delta) and minor point 1 (transcript pre-filtering). In both cases, Figures and Tables have been added as Supplementary material.*

Major points:

1.1 The prior on δ

The default prior (pages 3-4) on the parameters of the Dirichlet distribution is based on data pre-processing using another quantification tool (DRIMSeq). Although the authors do mention that δks could follow vaguely informative priors, I’ve found the adopted approach overly informative. Ideally, the authors should include in all of their comparisons and reported results (including diagrams and tables) the results of BANDITS arising from vaguely informative priors. This will give an idea to the reader about the sensitivity of the method with respect to prior choices.

*REPLY: We have run BANDITS with vaguely informative priors in all 3 simulated and 2 experimental data analyses. To avoid confusion (particularly in the plots), we did not add these results in the main manuscript Figures and Tables; instead, we created Supplementary Tables S11-S12 and Figures S18-S22 where results from BANDITS with informative and vaguely informative priors are compared. We comment about these results in the main text at page 8, right column, lines 1-16.*

1.2 Normalization with respect to effective length

The authors state that using the specific normalization (that is, using π ̄kT ’s instead of π ̄k’s) leads to improved performance when testing for DTU. Since all other methods are actually testing for differences in the latter, it would make sense to evaluate all methods on a common basis, that is, π ̄k’s. This will give a precise idea about the ability of each method to detect differences on a commonly used definition of what constitutes a ‘‘difference’’. Then, the authors can argue about using the specific normalization based on π ̄kT ’s, focusing on the enhanced performance of their proposed method.

*REPLY: While most methods test for differences in the parameter pi, there is a variety of measures that are used for testing by the methods considered. In particular, BANDITS uses the (normalized) parameter pi_T for testing, the 3 DEXSeq methods test the significance of a (group) interaction term in a negative-binomial model, SUPPA2 is based on percent-spliced-in estimates while limma works with linear models.*

*Nevertheless, in order to understand the actual gain provided by testing the normalized parameter pi_T, we modified the BANDITS package to test the original pi parameter and applied this modified version on the 3 simulated datasets. Supplementary Figures S25-S26 compare results obtained from BANDITS (testing pi_T) and its modified version (testing pi). A comment was also added in the main manuscript at page 4, right column, lines 22- 28.*

2 Minor comments

2.1 Transcript pre-filtering

The authors propose to use estimated counts in order to filter low expressed transcripts. Could you comment on the specific quantification choice (Salmon)? For example: how sensitive are the results if another quantification tool is used?

*REPLY: In all results presented in the previous version of the manuscript, reads were aligned with STAR and Salmon was used, on the aligned reads, to estimate transcript abundance and compute the equivalence classes and respective counts. We have now re-aligned the 6 vs. 6 simulation with Salmon directly (avoiding STAR alignment) and with kallisto, and performed inference with BANDITS (with and without pre-filtering transcripts).*

*Supplementary Figures S23-24 compare results of BANDITS on each three input data (STAR+Salmon, Salmon and kallisto); a comment about this was also added in the main text at page 8, right column, lines 17-29.*

*We find that aligning reads with Salmon and STAR provides similar results from BANDITS, while kallisto leads to increased FDRs for approximately the same TPR, particularly for gene-level testing.*

2.2 Computational benchmark

Briefly discuss the amount of memory used by each method.

*REPLY: This issue was also raised by Reviewer 2 (minor point 4): we report the same answer we wrote above.*

*To address this, we have extended our computational benchmark to also measure the maximum RAM used by each method.*

*The new results are described in the main (page 8, left column, lines 4-13), and shown in Supplementary Table S8 and Figure S12.*

**Second round of review**

**Reviewer 1**

The authors have addressed my comments in a careful and satisfactory manner.

**Reviewer 2**

The authors have provided additional detail as requested.  My previous comments have been satisfactorily addressed with the exception of the point below:

The Browser view given for Figure S17 does not adequately show a clear example of differential splicing.  The legend refers to three isoforms - UTP23-206, UTP23-203, and UTP23-207.  One exon of the annotated gene model for the UTP23-203 transcript model is shown, and none of the coverage tracks correspond to this isoform, since they all include exons that are excluded in UTP23-203.  UTP23-207 is not shown/labelled at all in the figure.  Additionally, the gene represents a likely difference in transcription termination.  The downstream exons are excluded in the control samples because they are not in the parent mRNA, not because of an alternative splicing path. This gene might be more appropriately labelled DTU rather than differential splicing.

The view presented in Fig S14/S15 is not clear, because as presented, the coverage shown in the differential region may be attributed to MRPS22 on the opposite strand.

**Reviewer 3**

The authors have submitted an improved version of their manuscript. Now, supplementary tables S11-S12, Figures S18-S22 and S23-S26 provide further insights to the reader regarding the sensitivity to prior assumptions and choice of the alignment and quantification tools.

I've found the latter of minor importance compared to the first one: it really seems that an informative prior is crucial for boosting the performance of BANDITS. Therefore, the following statement in the abstract of the paper:
"BANDITS has extremely favourable performance with respect to the competitors considered"
should be rephrased as
"BANDITS has extremely favourable performance with respect to the competitors considered, provided that an informative prior distribution is being used."

Minor comments:
Figure 2: state in the caption that not all tools guarantee FDR control in the transcript-level (as in the main text, page 5, right-column, line 37-39).

**Authors Response**

**Point-by-point responses to the reviewers’ comments:**

Reviewer #2:

The authors have provided additional detail as requested. My previous comments have been satisfactorily addressed with the exception of the point below:

COMMENT: The Browser view given for Figure S17 does not adequately show a clear example of differential splicing. The legend refers to three isoforms - UTP23-206, UTP23-203, and UTP23-207. One exon of the annotated gene model for the UTP23-203 transcript model is shown, and none of the coverage tracks correspond to this isoform, since they all include exons that are excluded in UTP23-203. UTP23-207 is not shown/labelled at all in the figure.

*REPLY: The browser view does not always allow to clearly observe the differential usage of all transcripts; therefore, we only focused on the most visible differentially used transcripts. To clarify this aspect, in Supplementary Section S1.5 we state that only “... some of these differences can also be easily visualized on the IGV plots, particularly those involving transcripts that are almost only expressed in one group.”. Nevertheless, to facilitate the visualization of the genome tracks, we now highlight, via red circles, the differentially used transcripts and the regions displaying the clearest difference between conditions (Supplementary Figures S14-S15 and S17-S18). Furthermore, we have added an additional image (Supplementary Figure S18) to show the junction tracks of the entire transcripts ENST00000517820 (UTP23-203) and ENST00000521703 (UTP23-207).*

COMMENT:

Additionally, the gene represents a likely difference in transcription termination. The downstream exons are excluded in the control samples because they are not in the parent mRNA, not because of an alternative splicing path. This gene might be more appropriately labelled DTU rather than differential splicing.

*REPLY: Thanks for the comment; we agree that the differences detected should be labelled as DTU instead of differential splicing. We have edited the text in Supplementary Section S1.5 and in the captions of Supplementary Figures S13-S18: we replaced “differentially spliced” with “differentially used”, and “differential splicing” with “differential usage”.*

COMMENT: The view presented in Fig S14/S15 is not clear, because as presented, the coverage shown in the differential region may be attributed to MRPS22 on the opposite strand.

*REPLY: Even though transcripts MRPS22-205 and COPB2-203 are partially overlapping, from the zoomed image in Figure S15, one can see that the junction reads in the red circle are only compatible with COPB2-203; we have now added red circles to facilitate the visualization of the image.*

Reviewer #3: The authors have submitted an improved version of their manuscript. Now, supplementary tables S11-S12, Figures S18-S22 and S23-S26 provide further insights to the reader regarding the sensitivity to prior assumptions and choice of the alignment and quantification tools.

COMMENT: I've found the latter of minor importance compared to the first one: it really seems that an informative prior is crucial for boosting the performance of BANDITS. Therefore, the following statement in the abstract of the paper:

"BANDITS has extremely favourable performance with respect to the competitors considered" should be rephrased as "BANDITS has extremely favourable performance with respect to the competitors considered, provided that an informative prior distribution is being used."

*REPLY: Thanks for your comment; however, we respectfully disagree with this wording. Often in Bayesian statistics, an informative prior distribution is obtained from other studies or datasets, which might or might not be available. However, the sentence “provided that an informative prior distribution is being used” actually suggests that an informative prior might not be available. Nonetheless, BANDITS infers an informative prior (for the precision parameter) from the available data (which is a very mild Empirical prior); hence, BANDITS will always be able to infer an informative prior and, therefore, it is always the case in BANDITS that “an informative prior distribution is being used”. Our approach is similar to the moderated estimation of the precision/dispersion parameters that is performed by differential methods such as edgeR, DESeq, DESeq2 or DRIMSeq. In these methods, the genewise estimates are shrunk towards a common trend, which is itself inferred from the available data.*

COMMENT:

Minor comments: Figure 2: state in the caption that not all tools guarantee FDR control in the transcriptlevel (as in the main text, page 5, right-column, line 37-39).

*REPLY: Many thanks for noticing this. We edited the caption of Figure 2 and of Supplementary Figure S2 and S8, to clarify that cjBitSeq transcript-level test does not guarantee FDR control.*
